# The Use of a Brief Synchronization Treatment after Weaning, Combined with Superovulation, Has Moderate Effects on the Gene Expression of Surviving Pig Blastocysts

**DOI:** 10.3390/ani13091568

**Published:** 2023-05-08

**Authors:** Henar Gonzalez-Ramiro, Maria A. Gil, Cristina Cuello, Josep M. Cambra, Alejandro Gonzalez-Plaza, Juan M. Vazquez, Jose L. Vazquez, Heriberto Rodriguez-Martinez, Alejandro Lucas-Sanchez, Inmaculada Parrilla, Cristina A. Martinez, Emilio A. Martinez

**Affiliations:** 1 Department of Medicine and Animal Surgery, Faculty of Veterinary Medicine, International Excellence Campus for Higher Education and Research, University of Murcia, 30100 Murcia, Spain; hgr@agropor.com (H.G.-R.); mariagil@um.es (M.A.G.); ccuello@um.es (C.C.); josepmiquel.cambra@um.es (J.M.C.); alejandro.gonzalez9@um.es (A.G.-P.); vazquez@um.es (J.M.V.); josel.vazquez@um.es (J.L.V.); emilio@um.es (E.A.M.); 2 Institute for Biomedical Research of Murcia (IMIB-Pascual Parrilla), Campus de Ciencias de la Salud, 30120 Murcia, Spain; 3 Department of Research and Development, Grupo Agropor I+D+I, AIE, 30565 Murcia, Spain; als@agropor.com; 4 Department of Biomedical & Clinical Sciences (BKV), BKH/Obstetrics & Gynecology, Faculty of Medicine and Health Sciences, Linköping University, 58185 Linköping, Sweden; heriberto.rodriguez-martinez@liu.se; 5 Department of Animal Reproduction, National Institute for Agriculture and Food Research and Technology (INIA-CSIC), 28040 Madrid, Spain

**Keywords:** Altrenogest, superovulation, estrus synchronization, embryo transcriptome, blastocyst, porcine

## Abstract

**Simple Summary:**

The use of combined estrus synchronization/superovulation (SS) treatments alters ovarian and endometrial gene expression patterns, impairing follicle and oocyte growth, fertilization, and embryo development. Since the impact of SS treatments on the transcriptome of the surviving embryos remains unidentified, we examined gene expression changes in day 6 blastocysts that survived a brief regimen of synchronization treatment combined with superovulation. Embryos were surgically collected on day 6 after AI, and transcriptome analysis was performed on blastocyst-stage embryos with good morphology to identify differentially expressed genes (DEGs) between groups at *p*-value < 0.05 and </>1.5-fold change. Compared with the blastocysts from control (untreated) sows, the blastocysts from SS-treated sows had moderate gene expression changes, with 7 pathways disrupted and a total of 10 transcripts affected, including up-regulation of metabolic *RDH10* and *SPTLC2* genes and down-regulation of oxidation-related *GSTK1* and *GSTO1* genes. These gene expression alterations may suggest suboptimal embryo quality and could depress the embryos’ response to oxidative stress, thereby impairing subsequent embryo development. These and previous findings call for the avoidance of SS treatments in embryo transfer programs.

**Abstract:**

The combination of estrus synchronization and superovulation (SS) treatments causes alterations in ovarian and endometrial gene expression patterns, resulting in abnormal follicle and oocyte growth, fertilization, and embryo development. However, the impact of combined SS treatments on the transcriptome of the surviving embryos remains unidentified. In this study, we examined gene expression changes in day 6 blastocysts that survived a brief regimen of synchronization treatment combined with superovulation. The sows were included in one of three groups: SS7 group (*n* = 6), sows were administered Altrenogest (ALT) 7 days from the day of weaning and superovulated with eCG 24 h after the end of ALT treatment and hCG at the onset of estrus; SO group (*n* = 6), ALT nontreated sows were superovulated with eCG 24 h postweaning and hCG at the onset of estrus; control group (*n* = 6), weaned sows displaying natural estrus. Six days after insemination, the sows underwent a surgical intervention for embryo collection. Transcriptome analysis was performed on blastocyst-stage embryos with good morphology. Differentially expressed genes (DEGs) between groups were detected using one-way ANOVA with an un-adjusted *p*-value < 0.05 and a fold change </> 1.5. The effect of SO treatment on the number of altered pathways and DEGs within each pathway was minimal. Only four pathways were disrupted comprising only a total of four altered transcripts, which were not related to reproductive functions or embryonic development. On the other hand, the surviving blastocysts subjected to SS7 treatments exhibited moderate gene expression changes in terms of DEGs and fold changes, with seven pathways disrupted containing a total of 10 transcripts affected. In this case, the up-regulation of certain pathways, such as the metabolic pathway, with two up-regulated genes associated with reproductive functions, namely *RDH10* and *SPTLC2*, may suggest suboptimal embryo quality, while the down-regulation of others, such as the glutathione metabolism pathway, with down-regulated genes related to cellular detoxification of reactive oxygen species, namely *GSTK1* and *GSTO1*, could depress the embryos’ response to oxidative stress, thereby impairing subsequent embryo development. The gene expression changes observed in the present study in SS7 embryos, along with previous reports indicating SS7 can negatively affect fertilization, embryo production, and reproductive tract gene expression, make its use in embryo transfer programs unrecommendable.

## 1. Introduction

Recent advancements made porcine nonsurgical embryo transfer (Ns-ET) with fresh and vitrified embryos possible [1,2,3,4,5,6,7], enabling the wider use of this highly demanded technology by the swine industry. However, one of the problems that still persists is the high number of embryos (>25) required for each ET, which determines a donor:recipient ratio greater than 2:1 [8] increasing the cost per ET. Superovulation with gonadotropins of previously synchronized donor sows through weaning is effective in reducing this ratio. Weaning is the most useful natural method for estrus synchronization allowing sows to be grouped efficiently, which simplifies embryo collection. In weaned sows, superovulation treatment provided excellent results by increasing the number of high-quality embryos, without affecting fertilization rates [9] or post-Ns-ET fertility and prolificacy [9,10]. Despite the effectiveness of this weaning–superovulation treatment, ET programs often need a larger number of donors than a single weaning can offer, requiring several weanings to provide enough donors for each ET batch.

There is evidence that postweaning estrus is retarded by the short-term (St) application of the progestogen Altrenogest (ALT), which makes it possible to synchronize the onset of the postweaning estrus from multiple weanings without adverse impact on ovulation and embryonic development [11,12]. However, the situation becomes very different when superovulation is induced after the St-ALT treatment. Recently, we have shown that St-ALT administration before superovulation was efficient in delaying postweaning estrus and increasing the ovulation rates in weaned sows, but the treatments decreased fertilization, pregnancy, and embryo production rates compared to ALT-untreated, superovulated sows [13,14]. The poor results achieved after the combined use of St-ALT and superovulation treatments were mainly due to the increased proportions of immature oocytes and degenerated embryos compared to those obtained in ALT-untreated, superovulated sows and sows with natural estrus after weaning. It has recently been shown that the combination of these treatments alters the gene expression patterns in the ovary and endometrium, mainly dysregulating key metabolic pathways, which could lead to inadequate follicle and oocyte growth and preimplantation embryo developmental ability [14]. Although the quality of the surviving blastocysts after St-ALT plus superovulation treatment, in terms of total trophectoderm and inner cell mass cells, apoptosis rates, and cryotolerance [13], was similar to that observed in ALT-untreated, superovulated sows and in natural postweaning estrus sows, the treatment can influence not only oocyte maturation and fertilization but also surviving embryos at the molecular level. The aim of the present investigation was to establish the extent to which St-ALT treatment in combination with a standard superovulation protocol in weaned sows caused variations in the overall transcriptome of day 6 embryos.

## 2. Materials and Methods

The substances employed in the research were obtained from Sigma-Aldrich Química S.A. (Madrid, Spain), unless specified otherwise.

### 2.1. Animals and Ethics

The study was performed on a farm located in Murcia, Spain (Agropor SL). Landrace × Large White sows with lactation lengths of 24–28 days (mean ± SD: 24.9 ± 1.4 days) were arbitrarily selected at weaning and individually placed in a barn with controlled humidity and temperature. Average daily temperature and temperature–humidity index in the barn ranged from 14.6 °C to 24.0 °C and from 58.2 to 67.83, respectively. Two mature boars allocated in an artificial insemination (AI) center (AIM Iberica, Murcia, Spain) delivered semen to prepare AI seminal doses. The sows and boars had water freely available and were fed in concordance with their nutritional necessities [15,16]. The research was granted approval by the Ethics Committee for Animal Experimentation of the University of Murcia (date: 22 November 2018; code: 486/2018).

### 2.2. Experimental Design

After weaning, sows were selected based on their body condition score (>2.7 on a 5-point scale), parity (ranging from 4 to 7), prior fertility (>80%), and prolificacy (>11.0 piglets), with no differences among the groups.

Animals were included in one of three groups: SS7 group (*n* = 6), sows were administered ALT (Regumate, Merck Sharp & Dohme Animal Health, S.L., Salamanca, Spain) 7 days from the day of the weaning and superovulated with eCG (Foligon, Intervet, Boxmeer, the Netherlands) 24 h after the end of ALT treatment and hCG (Veterin Corion, Divasa, Farmavic S.A., Barcelona, Spain) at the onset of estrus; SO group (*n* = 6), ALT nontreated sows were superovulated with eCG 24 h postweaning and hCG at the onset of estrus; control group (*n* = 6), weaned sows displaying natural estrus. In three replicates, 18 sows (6 sows per group) were inseminated with AI doses from the same boar. At day 6 of the cycle (day 0 = onset of estrus), the sows underwent surgery for embryo collection. Transcriptome analysis was performed on early and full blastocyst-stage embryos with excellent or good morphology. These embryonic stages were equally distributed among the three groups. Five blastocysts from each sow of each group were placed into a tube containing 5 µL of PBS, snap-frozen in liquid nitrogen, and stored at −80 °C until further analysis. We conducted 18 microarray analyses (6 for each group), and the outcomes were authenticated via quantitative real-time polymerase chain reaction (RT-qPCR), which involved three biological and three technical replicates for each gene.

### 2.3. Treatments, Detection of Estrus, and Insemination

To delay the postweaning estrus, sows were orally administered 20 mg/sow/day of ALT. Superovulation was induced in the sows via intramuscular injection of 1000 IU eCG and 750 IU hCG. Estrus detection was carried out by trained personnel once daily, commencing on the day of the final ALT administration or on the day of weaning depending on the groups. AI was conducted at 6 h and 24 h from the onset of estrus using 3 × 10^9^ spermatozoa in a 90 mL BTS extender [17]. All AI doses exhibited more than 75% sperm motility and less than 20% morphological sperm abnormalities at the time of AI.

### 2.4. Collection of Embryos

The surgical collection of embryos was conducted in accordance with the previously described protocol [18]. In brief, after administering sedation (azaperone; Stresnil, Landegger Strasse, Austria), the sows were anesthetized with sodium thiopental (B. Braun VetCare SA, Barcelona, Spain) and then maintained under isoflurane (IsoFlo, Madrid, Spain). During laparotomy, the ovaries were inspected to count the number of corpora lutea in each sow, and the embryos were retrieved from the uterus using 30 mL of TL-HEPES medium [7,19] at 38 °C.

### 2.5. Embryo Quality Evaluation

The quality and stage of embryo development were determined by stereomicroscopy. Structures with a single cell were considered oocytes. Embryos insufficiently developed or with inadequate morphology were classified as degenerate. Embryos with proper developmental stage and adequate morphology according to IETS criteria [20] were classified as viable and transferable.

### 2.6. Total RNA Extraction

The RNeasy Micro Kit (Qiagen Iberica, Madrid, Spain) was used for the extraction of total RNA from blastocysts following the manufacturer’s manual. The concentration and quality of the extracted RNA were determined with the 2100 Bioanalyzer (Agilent, Santa Clara, CA, USA). The RNA integrity Number (RIN) of each sample was greater than 8.5.

### 2.7. Microarray Processing

Gene expression differences among groups were determined with the GeneChip Porcine Gene 1.1 ST Array Strip (901798, Affymetrix, Thermo Fisher Scientific, Waltham, MA, USA), which contains 394,850 probes for the identification of 19,212 genes and covers the transcriptome of Sus scrofa. ss-cDNA was produced from 800 pg or total RNA of each sample using the GeneChip WT Pico Reagent Kit (P/N 902623; Thermo Fisher Scientific, Waltham, MA, USA) following the recommendations of the manufacturer. The amount and quality of cDNA were checked by Nanodrop and Bioanalyzer. ss-cDNA targets were cleaned up and after fragmentation and terminal labeling, and 3.75 µg of fragmented and biotinylated ss-cDNA were included in the hybridization mix, using the GeneAtlas Hybridization, Wash and Stain kit for WT Array Strips (Affymetrix, P/N 901667) according to recommendations of the manufacturer. The resulting preparations were hybridized to the array strip (Porcine Gene 1.1 ST Array Strip) for 20 h at 48 °C. After incubation, arrays were washed, stained, and scanned using GeneAtlas System (Affymetrix, Thermo Fisher Scientific, Waltham, MA, USA). The data were then analyzed using Affymetrix’s Expression Command Console, and all samples passed the quality criteria.

### 2.8. Analysis of the Microarray Data

The data underwent normalization using the Robust Multichip Average (RMA) method [21], with background correction followed by quantile normalization to generate a value for each probeset. Statistical and functional interpretation of data was conducted using the software of the Partek Genomics Suite and Partek Pathways (Partek Incorporated, St. Louis, MO, USA). Principal component analysis (PCA) was utilized as an exploratory tool to examine transcriptome variations among the samples. Differentially expressed genes (DEGs) between groups were detected using one-way ANOVA with an un-adjusted *p*-value < 0.05 and a fold change </> 1.5. The identification of overrepresented gene sets (Gene Ontology and pathway categories) in the DEG list was based on the Kyoto Encyclopedia of Genes and Genomes (KEGG) database [22] using Fisher’s exact test.

### 2.9. Real-Time, Reverse Transcription Quantitative PCR (RT-qPCR) Assay

A total of 7 DEGs, 5 up-regulated (*CHSY1*, *CSGALNACT2*, *CTPS1*, *RDH10*, *SPTLC2*) and 2 down-regulated (*GSTK1*, *ATG4C*) were examined by RT-qPCR to confirm the microarray results using the same RNA extracted for the analysis of microarrays. cDNA was generated using the Maxima H Minus First Strand cDNA Synthesis Kit (Thermo Fisher Scientific, Waltham, MA, USA) with successive incubations of 10 min, 15 min, and 5 min at 25 °C, 50 °C, and 85 °C, respectively. The Primer Express software v3.0.1 (Applied Biosystems, Foster City, CA, USA) was used to design the primers, which were commercially synthesized (Table 1). The assay of RT-qPCR was carried out with iTaq Universal SYBR Green Supermix (Applied Biosystems, Foster City, CA, USA) in 10 μL (including 2 μL cDNA and 500 nM of each primer). A QuantStudio 5 Real-Time PCR System (Applied Biosystems) was used for the reactions. Cycles consisted of 2 min at 50 °C for Uracil-DNA glycosylase activation, 10 min at 95 °C for initial denaturation, and 40 cycles of 15 s at 95 °C and 1 min at 60 °C.

Each RT-qPCR’s specificity was assessed by performing a melt curve analysis, which was incorporated into each PCR reaction. The expression levels of each gene were quantified as previously reported [23] and normalized to the housekeeping reference genes *ACTB* (Actin Beta) and *MRPL46* (Mitochondrial Ribosomal Protein L46). The RT-qPCR data analysis was carried out using IBM SPSS Statistics package version 28.0.1.1 (IBM, Chicago, IL, USA), with statistical significance considered at a *p*-value less than 0.05 and evaluated using Student’s *t*-test.

## 3. Results

Data on embryo recovery, fertilization, and embryo viability in animals from SS7, SO, and control groups have been previously reported [14]. The interval from weaning to estrus for the SO and control sows was 4.2 ± 0.4 and 4.3 ± 0.5 days, respectively, and the interval from ALT withdrawal to estrus was 4.8 ± 0.4 days, with no differences among groups. The number of embryos collected from each sow in the three groups and the embryo developmental stage and quality are shown in Table 2. These embryos, at the blastocyst stage, were used for transcriptome analysis.

### 3.1. Gene Expression Profiles of Embryos

Analysis of the transcriptome changes of embryos after SS7 and SO treatments showed 172 and 97 transcripts being differentially expressed in embryos from SS7 and SO groups relative to control embryos. Among these, 85 (48 and 37 up- and down-regulated, respectively) and 36 (8 and 28 up- and down-regulated, respectively) transcripts were annotated as known genes in the SS7 embryos (Supplementary Table S1) and SO embryos (Supplementary Table S2), respectively. More than 50% of transcripts were not annotated.

**Figure 1:**
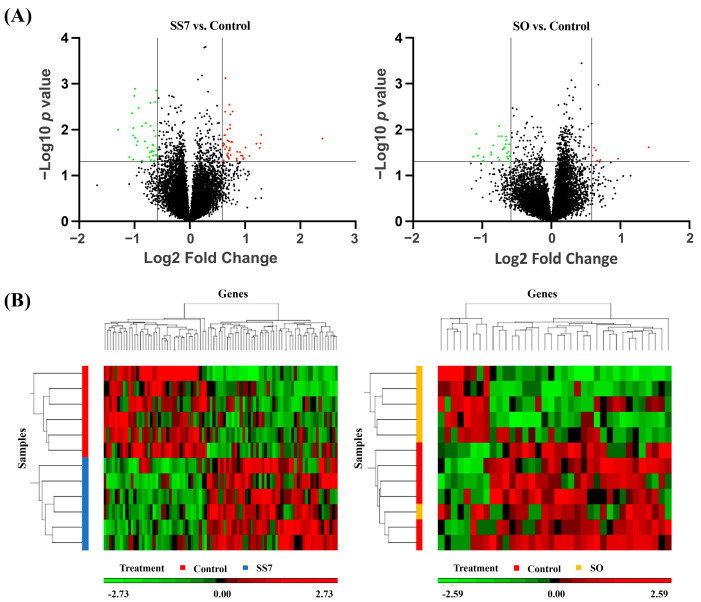
Volcano plots (**A**) and heat maps (**B**) comparing differentially expressed genes between synchronized–superovulated (SS7) and control embryos and between superovulated (SO) and control embryos. The colors denote different levels of gene expression levels (red, up-regulation; green, down-regulation).

PCA over all genes split SS7 and SO from control samples. The first three PCA axes accounted for 51.6% (SS7 vs. control) and 50.1% (SO vs. control) of data variance, with the first axis accounting for 24.3% and 25.3%, respectively. The DEGs detected in SS7 and SO blastocysts relative to the control blastocysts are shown in a volcano plot (Figure 1A). Based on hierarchical clustering, the DEGs between SS7 embryos and control embryos were strikingly segregated into two groups, but this segregation was not evident for the DEGs between SO embryos and control embryos (Figure 1B).

### 3.2. GO and Pathway Enrichment of DEGs

**Figure 2:**
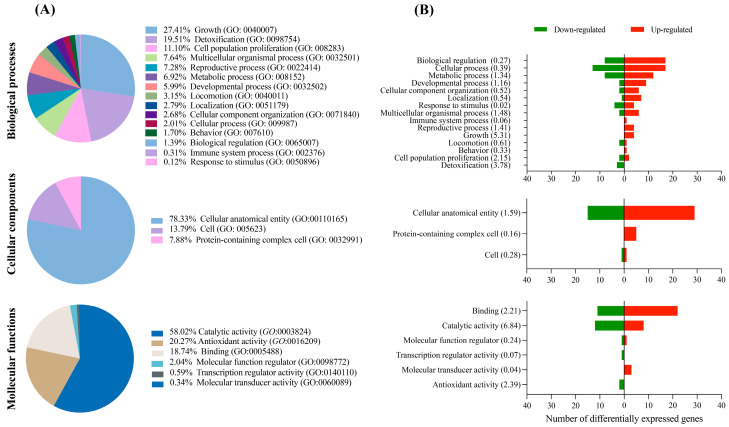
Functional enrichment analysis. (**A**) Gene Ontology analysis (KEGG database) of the differentially expressed genes between embryos collected from synchronized–superovulated (SS7) and control (untreated) sows. (**B**) Number of up- and down-regulated genes in each GO category; the enrichment score is shown in parentheses.

**Figure 3:**
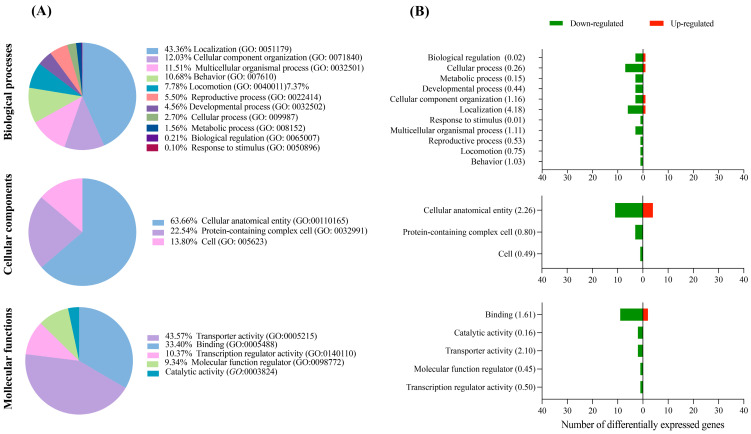
Functional enrichment analysis. (**A**) Gene Ontology analysis (KEGG database) of the differentially expressed genes between embryos collected from superovulated (SO) and control (untreated) sows. (**B**) Number of up- and down-regulated genes in each GO category; the enrichment score is shown in parentheses.

According to the KEGG database, the DEGs fit into three main GO categories: biological processes, cellular components, and molecular functions. While the majority of the DEGs in the SS7 embryos were associated with biological processes such as cell growth, detoxification, and proliferation of cell populations (Figure 2A), DEGs from SO embryos were related to localization, cellular component organization, and multicellular organismal process (Figure 3A). Cell anatomical entity was the most highly represented category within cellular components for both SS7 and SO embryos (Figure 2A and Figure 3A, respectively). Genes involved in molecular functions such as catabolic activity, antioxidant activity, and binding (SS7 embryos) or binding and transporter activity (SO embryos) were the most abundant (Figure 2A and Figure 3A). Generally, more up-regulated and down-regulated genes were detected in SS7 and SO embryos, respectively, in each biological function (Figure 2B and Figure 3B).

To identify significant KEGG pathways, dataset lists including all DEGs (ALL), up-regulated DEGs (UP), and down-regulated DEGs (DOWN) were considered. In the SS7 treatment, we found six, two, and five pathways enriched in the ALL, UP, and DOWN lists, respectively, resulting in eight pathways that were specifically affected by the treatment (Table 3). In the SO treatment, only two, one, and three pathways were enriched in the ALL, UP, and DOWN lists, respectively, with a total of four pathways influenced by the treatment (Table 4).

KEGG pathway analysis in the UP list of SS7 vs. control embryos showed enrichment of pathways associated with glycan biosynthesis (glycosaminoglycan biosynthesis—chondroitin sulfate/dermatan sulfate) and metabolic pathways, and these pathways comprised genes (*CHSY1*, *CSGALNACT2*, *CTPS1*, *RDH10*, and *SPTLC2*) that affect main reproductive processes (Table 4). Six pathways, metabolism of xenobiotics by cytochrome P450, drug metabolism—cytochrome P450, glutathione metabolism, chemical carcinogenesis, lysosome, and terpenoid backbone biosynthesis, were enriched in the DOWN list of SS7 vs. control embryos and contained genes (*GSTK1*, *GSTO1*, *ASAH1*, *SUMF1*, and *FDPS*) with key roles in cell functions (Table 5).

Comparison between SO embryos and control embryos provided only one (natural killer cell-mediated cytotoxicity) and three (collecting duct acid secretion, autophagy, and N-glycan biosynthesis) enriched pathways in the UP and DOWN lists, respectively, and these pathways contained genes (*HCST*, *ATP6V1B1*, *ATG4C*, and *MAN1A2*) seemingly unrelated to reproduction (Table 6).

### 3.3. Microarray Validation

**Figure 4:**
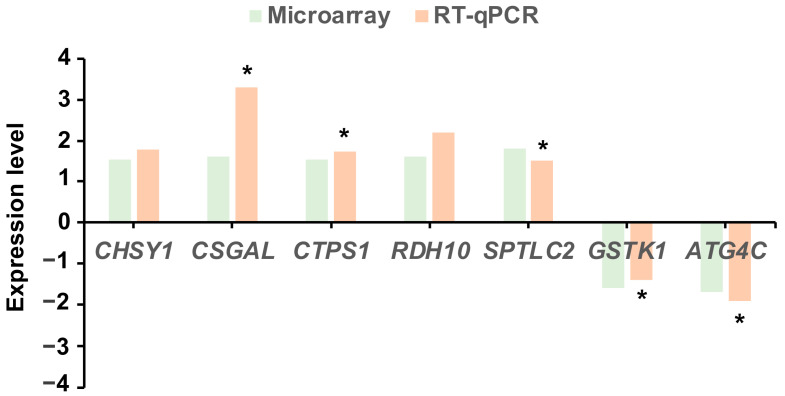
Comparison of microarray and real-time quantitative PCR (RT-qPCR) data of seven differentially expressed genes determined by microarray analysis. Data are expressed as fold change of gene expression of embryos derived from synchronized–superovulated or superovulated groups and control groups. * Differences (*p* < 0.05) among experimental and control groups as determined by RT-qPCR.

We selected seven genes to validate the microarray results using RT-qPCR. Three up-regulated and two down-regulated genes showed comparable significant results with the PCR method. The other two genes presented the same pattern with both methods but without significant differences between groups when analyzed by RT-qPCR (Figure 4). These results support the reliability of the microarray results.

## 4. Discussion

To the best of our knowledge, this is the first study investigating the effects of a combined treatment of synchronization and superovulation (SS7) on gene expression of porcine day 6 embryos. Our findings suggest that the impact of the treatment on the number and fold changes of DEGs in blastocysts was moderate compared to control embryos obtained from weaned sows with natural estrus.

The gene expression patterns of SO embryos were comparable to those of the control embryos with only 36 DEGs between both groups and only 5 of them with a fold change greater than 2. The impact of that treatment on the number of altered pathways and the proportion of DEGs in each pathway was negligible. Moreover, these DEGs were not associated with embryonic development or other reproductive processes. These results were not unexpected, since several studies have shown that the superovulation treatment utilized in this investigation increased the number of transferable day 6 blastocysts without any impact on the reproductive performance of the recipients following the nonsurgical ET of those superovulated embryos [9,13]. Therefore, we can assume that superovulation of weaned sows is a productive approach to increase the number of transferable embryos, without altering their in vivo developmental ability, in the ET programs.

In contrast, when this superovulation treatment is combined with a previous estrus synchronization short treatment, the percentage of day 6 viable embryos is dramatically decreased as a result of the substantial rise in the percentages of both immature oocytes ovulated and degenerated embryos [13,14]. However, our results suggest that the embryos surviving this combined hormonal treatment present moderate changes in gene expression when compared to the controls. We identified 85 DEGs, from which only 14 DEGs had a fold change greater than 2. Nevertheless, this number of DEGs, although low, may be related to gross embryological changes. Two facts support this observation. First, morphologically degenerative embryos only presented 47 DEGs compared with normal embryos [24]; second, although vitrification adversely affects pig embryo quality [25,26] and decreases subsequent in vivo embryonic development after ET [27], minor gene expression changes between vitrified and fresh embryos have been reported [28,29].

The KEGG pathway enrichment analysis of DEGs in SS7 embryos revealed that the treatment had a moderate impact in terms of the number of pathways that were altered and the proportion of DEGs within each pathway. Two pathways, the glycosaminoglycan biosynthesis—chondroitin sulfate/dermatan sulfate pathway and the metabolic pathway, were up-regulated. Chondroitin/dermatan sulfate (CS/DS) is a group of glycosaminoglycans (GAGs) that are present on the surfaces of almost all cells and within extracellular matrices. They have a crucial function in multiple biological processes, such as interacting with diverse growth factors, regulating cell proliferation and differentiation, facilitating cell adhesion, and contributing to tissue morphogenesis [30,31]. Moreover, early embryonic cell division in mammals relies on the presence of CS, which is produced through the activity of glucuronyltransferase-I (GT-I). Knock-out mice lacking this enzyme resulted in a significant decrease in CS synthesis ultimately causing embryonic lethality before the eight-cell stage [32].

Interestingly, the levels of GAGs in the extracellular matrix of the endometrium and myometrium of rabbits and rats are influenced by hormonal balance, increasing under progestogen administration [33,34]. Considering this information, we hypothesize that the short progestogen treatment used in our study could be responsible for the enrichment of the GAG biosynthesis pathway in the SS7 embryos. Two transcripts showed an increase in activity in that pathway, *CHSY1* and *CSGALNACT2*. Both transcripts, which encode glycosyltransferases that participate in the biosynthesis of CS, are recognized as significant regulators in different biological processes. Specifically, *CHSY1* is associated with bone development and digit patterning, while *CSGALNACT2* plays a role in pulmonary and skeletal development during embryogenesis [31,35]. The specific importance of the up-regulation of these transcripts in the developmental ability of SS7 embryos is uncertain, but it could be exclusively related to the response of these embryos to the hormonal stress caused by the synchronization–superovulation treatment. The enrichment of the metabolic pathway may indicate poor embryo quality. If the “quiet embryo” theory is considered, which suggests that preimplantation embryo survival is linked to a relatively subdued metabolism, the up-regulation of this pathway might negatively affect the development and implantation of SS7 embryos [36]. Gene expression analysis showed that SS7 embryos had higher levels of metabolism, which, considering previous studies [37], should be associated with lower embryo survival. Among the up-regulated genes in that pathway, *RDH10* and *SPTLC2* have been linked to various reproductive processes. *RDH10* is a crucial gene for the production of retinoic acid (RA) during embryogenesis, which is involved in numerous biological functions, such as embryo development [38]. Studies in rodents have revealed that severe abnormalities and lethality at mid-pregnancy can occur when the metabolism of RA is restricted or interrupted during development [39,40]. However, excessive levels of RA during crucial developmental stages can lead to malformations or lethality in the embryo. [41]. Thus, the control of the appropriate quantity of RA available to the embryo at particular times and in specific tissue locations is crucial [38]. We observed an increase in *RDH10* gene expression in SS7 embryos, which might indicate a reduction in RA levels, as *RDH10* plays a vital role as a control point for providing feedback regulation of RA synthesis [42].

The gene SPTLC2 is a subunit of the serine palmitoyltransferase (SPT) holoenzyme, which is essential in sphingolipid metabolism [43]. In mice, the absence of this subunit leads to the cessation of enzymatic activity of the SPT, which is necessary for normal embryonic progress [44] because sphingolipids play a vital role as signaling molecules that govern numerous cellular processes [45,46]. Moreover, it appears that de novo synthesis of sphingolipids is necessary for successful mouse implantation, as indicated by the increased expression levels of *SPTLC2* at implantation sites [47]. Our study suggests that the up-regulation of the *SPTLC2* transcript in SS7 embryos might be a result of progestogen treatment used for estrus synchronization, given that progesterone has been shown to stimulate the expression of *SPTLC2* in the mouse uterus [47].

The analysis of KEGG pathways based on down-regulated DEGs in SS7 embryos indicated that three pathways, the metabolism of xenobiotics by cytochrome P450, drug metabolism—cytochrome P450, and glutathione metabolism, exhibited reduced expression levels. Only two genes, *GSTK1* and *GSTO1*, were found to be altered in these pathways.

The other two down-regulated pathways, the lysosome and terpenoid backbone biosynthesis pathways, had only three altered genes, *ASAH1*, *SUMF1*, and *FDPS*.

The *GSTK1* and *GSTO1* genes encode glutathione S-transferase (GST) proteins with important functions in cellular detoxification. The encoded proteins catalyze the conjugation of glutathione to hydrophobic substrates, thereby contributing to the elimination of these compounds from cells and protecting them from oxidative stress [48,49]. Down-regulation of these genes may impair metabolism, allowing a maladaptive response to oxidative stress and other toxic stimuli, and negatively impact subsequent embryo development, as suggested in studies on cryopreserved mouse embryos [50,51].

The lysosome and terpenoid backbone biosynthesis pathways were altered by the down-regulation of *ASAH1*, *SUMF1*, and *FDPS* genes. The *ASAH1* gene encodes acid ceramidase (AC), an important enzyme implicated in ceramide metabolism. Under normal circumstances, the levels of ceramide are low. However, in response to various stimuli, its production at the cell surface increases quickly, causing a reorganization of the membrane that ultimately leads to apoptosis. AC induces the hydrolysis of ceramide and the production of sphingosine-1-phosphate [52], and it is considered fundamental for embryo survival because it inhibits the default apoptosis pathway [53]. The remaining two genes were not associated with either embryonic development or implantation.

## 5. Conclusions

Together, our results show that the number of altered pathways and the number of DEGs within each pathway in day 6 blastocysts were minimally affected by the SO treatment. A total of four pathways were disrupted, with just four altered transcripts, which were not associated with embryo development or other reproductive processes. These results corroborate that the superovulation of weaned sows is an efficient technique to enhance the number of transferable embryos in ET programs. After SS7 treatment, the surviving blastocysts showed moderate gene expression changes in terms of DEGs and fold changes. A total of seven pathways were disrupted by SS7 treatment, comprising 10 altered transcripts. Up-regulation of certain pathways, such as the metabolic pathway, may indicate suboptimal embryo quality, while the down-regulation of the glutathione metabolism pathway could reduce the response of the embryos to oxidative stress, which would impair subsequent embryo development. Based on these and earlier results demonstrating a negative impact of SS7 on fertilization, embryo production, and ovarian and uterine gene expression, the utilization of SS7 in ET programs is discouraged. These findings are of great significance for the swine ET industry since they prevent the combined use of two hormonal treatments (synchronization and superovulation) that function effectively when applied separately. This, consequently, avoids a reduction in the reproductive potential of embryo donor sows.

## Figures and Tables

**Table 1:** Primer sequences for RT-qPCR and amplicon sizes.

Gene	Accession Number	Primers (5′-3′)	Size (pb)	Efficiency (%)	R^2^
*CHSY1*	NM_001244442.2	F: GAGATGCGTGCGAAGGTT R: GTAGGGTGGGTTCTTGTTGG	166	83.9	0.997
*CSGALNACT2*	XM_001926417	F: CTGCCATTGTTTATGCCAAT R: ATTCCAAAGCCAAAATCTCG	100	110.5	0.998
*CTPS1*	XM_003128105.4	F: GGAACATTCTCTCCCTATGAGC R: GTCGGATGTCAAGGAAACG	103	101.9	1.000
*RDH10*	XM_021089230.1	F: ACATCAACACGCAGAGCAAC R: CTCTCTTTCCCACATCACAGG	168	93.0	0.993
*SPTLC2*	XM_005656439.2	F: ATCTGCGGACACATTCTCAC R: CCTGGTGTTTTCGGCTAACT	152	102.6	0.999
*GSTK1*	NM_001315574.1	F: GCAGGAGAAAGGGAACGAT R: TGCAGGTTGACATTCCAGAT	183	116.5	0.996
*ATG4C*	NM_001190284.1	F: GTCGAAATGTTCAGGACTTCA R: GACAAACTCTTCTGTGCTAAATCTG	206	99.1	0.995
*ACTB*	XM_003357928	F: CTCGATCATGAAGTGCGACGT R: TGATCTCCTTCTGCATCCTGTC	114	101.8	0.998

**Table 2:** Effects of the combined treatment for estrus synchronization and superovulation in weaned sows on developmental stage and quality of the collected day 6 embryos.

Group	Sows (*n*)	Viable Embryos (*n*, Mean ± SD)	Oocytes/Degenerated Embryos (*n*, Mean ± SD)	Embryo Stage *	Embryo Quality ** (*n*, %)
SS7	6	132 (22.0 ± 3.9) ^a^	55 (9.2 ± 4.3) ^a^	2.4 ± 0.4	115 (87.1)
SO	6	174 (29.0 ± 1.8) ^b^	12 (2.0 ± 2.4) ^b^	2.5 ± 0.4	162 (93.1)
Control	6	105 (17.5 ± 4.7) ^a^	9 (1.5 ± 1.4) ^b^	2.5 ± 0.2	99 (94.3)

* The developmental stage was scored according to the following classes: 1: morulae; 2: early blastocysts; 3: blastocysts; 4: expanded blastocysts. ** Embryos with quality grade 1 or 2 (excellent or good morphology). Different superscripts in the same column indicate differences (*p* < 0.05) (ANOVA followed by Bonferroni post hoc test).

**Table 3:** Enrichment analysis of KEGG pathways in synchronized–superovulated (SS7) embryos compared to control embryos.

Pathway ID	Pathway Name	Enrichment *p*-Value *		
		All	Up	Down
ssc00532	Glycosaminoglycan biosynthesis—chondroitin sulfate/dermatan sulfate	**0.002**	**0.001**	-
ssc01100	Metabolic pathways	**0.002**	**0.007**	0.078
ssc00980	Metabolism of xenobiotics by cytochrome P450	**0.007**	-	**0.003**
ssc00982	Drug metabolism—cytochrome P450	**0.007**	-	**0.003**
ssc00480	Glutathione metabolism	**0.009**	-	**0.003**
ssc00600	Sphingolipid metabolism	**0.009**	0.053	0.088
ssc04142	Lysosome	0.055	-	**0.026**
ssc00900	Terpenoid backbone biosynthesis	0.066	-	**0.042**

* Enriched pathways with significant values in each gene list are shown in bold.

**Table 4:** Enrichment analysis of KEGG pathways in superovulated (SO) embryos compared to control embryos.

Pathway ID	Pathway Name	Enrichment *p*-Value *		
		All	Up	Down
ssc04966	Collecting duct acid secretion	**0.028**	**-**	**0.024**
ssc04136	Autophagy—other	**0.034**	**-**	**0.030**
ssc00510	N-Glycan biosynthesis	0.051	-	**0.045**
ssc04650	Natural killer cell-mediated cytotoxicity	0.102	**0.013**	-

* Enriched pathways with significant values in each gene list are shown in bold.

**Table 5:** Enriched (*p* < 0.05) KEGG pathways in embryos from synchronized–superovulated (SS7) sows compared with the control embryos.

Pathway ID	Pathway Name	Pathway Alteration	ES *	Altered Genes (%)	Gene List
ssc00532	Glycosaminoglycan biosynthesis—chondroitin sulfate/dermatan sulfate	Up-regulation	8.1	13.3	*CHSY1*, *CSGALNACT2*
ssc01100	Metabolic pathways	Up-regulation	4.9	0.6	*CHSY1*, *CSGALNACT2*, *CTPS1*, *RDH10*, *SPTLC2*
ssc00980	Metabolism of xenobiotics by cytochrome P450	Down-regulation	5.9	7.7	*GSTK1*, *GSTO1*
ssc00982	Drug metabolism—cytochrome P450	Down-regulation	5.8	7.4	*GSTK1*, *GSTO1*
ssc00480	Glutathione metabolism	Down-regulation	5.7	6.9	*GSTK1*, *GSTO1*
ssc04142	Lysosome	Down-regulation	3.6	2.4	*ASAH1*, *SUMF1*
ssc00900	Terpenoid backbone biosynthesis	Down-regulation	3.2	7.1	*FDPS*

* ES: enrichment score.

**Table 6:** Enriched (*p* < 0.05) KEGG pathways in embryos from superovulated (SO) sows compared with the control embryos.

Pathway ID	Pathway Name	Pathway Alteration	ES *	Altered Genes (%)	Gene List
ssc04650	Natural killer cell-mediated cytotoxicity	Up-regulation	4.3	1.0	*HCST*
ssc04966	Collecting duct acid secretion	Down-regulation	3.7	3.8	*ATP6V1B1*
ssc04136	Autophagy—other	Down-regulation	3.5	3.1	*ATG4C*
ssc00510	N-Glycan biosynthesis	Down-regulation	3.1	2.0	*MAN1A2*

* ES: enrichment score.

## Data Availability

The raw datasets generated during and/or analyzed during the current study are available in ArrayExpress accession number: E-MTAB-12820.

## Supplementary Materials

The following supporting information is available online at https://www.mdpi.com/article/10.3390/ani13091568/s1, Table S1: Differentially expressed transcripts in SS7 porcine blastocysts. Table S2: Differentially expressed genes in SO porcine blastocysts.
